# Properties of Experimental Dental Composites Containing Antibacterial Silver-Releasing Filler

**DOI:** 10.3390/ma11061031

**Published:** 2018-06-18

**Authors:** Robert Stencel, Jacek Kasperski, Wojciech Pakieła, Anna Mertas, Elżbieta Bobela, Izabela Barszczewska-Rybarek, Grzegorz Chladek

**Affiliations:** 1 Private Practice, Center of Dentistry and Implantology, ul. Karpińskiego 3, 41-500 Chorzów, Poland; robert.stencel@op.pl; 2 Department of Prosthetic Dentistry, School of Medicine with the Division of Dentistry in Zabrze, Medical University of Silesia, pl. Akademicki 17, 41-902 Bytom, Poland; kroczek91@interia.pl; 3 Faculty of Mechanical Engineering, Institute of Engineering Materials and Biomaterials, Silesian University of Technology, ul. Konarskiego 18a, 44-100 Gliwice, Poland; wojciech.pakiela@polsl.pl; 4 Chair and Department of Microbiology and Immunology, School of Medicine with the Division of Dentistry in Zabrze, Medical University of Silesia in Katowice, ul. Jordana 19, 41-808 Zabrze, Poland; amertas@sum.edu.pl (A.M.); ebobela@sum.edu.pl (E.B.); 5 Department of Physical Chemistry and Technology of Polymers, Silesian University of Technology, 44-100 Gliwice, Poland; Izabela.Barszczewska-Rybarek@polsl.pl

**Keywords:** dental composites, antibacterial properties, silver, mechanical properties, degree of conversion, sorption, solubility, color stability

## Abstract

Secondary caries is one of the important issues related to using dental composite restorations. Effective prevention of cariogenic bacteria survival may reduce this problem. The aim of this study was to evaluate the antibacterial activity and physical properties of composite materials with silver sodium hydrogen zirconium phosphate (SSHZP). The antibacterial filler was introduced at concentrations of 1%, 4%, 7%, 10%, 13%, and 16% (*w*/*w*) into model composite material consisting of methacrylate monomers and silanized glass and silica fillers. The in vitro reduction in the number of viable cariogenic bacteria *Streptococcus mutans* ATCC 33535 colonies, Vickers microhardness, compressive strength, diametral tensile strength, flexural strength, flexural modulus, sorption, solubility, degree of conversion, and color stability were investigated. An increase in antimicrobial filler concentration resulted in a statistically significant reduction in bacteria. There were no statistically significant differences caused by the introduction of the filler in compressive strength, diametral tensile strength, flexural modulus, and solubility. Statistically significant changes in degree of conversion, flexural strength, hardness (decrease), solubility (increase), and in color were registered. A favorable combination of antibacterial properties and other properties was achieved at SSHZP concentrations from 4% to 13%. These composites exhibited properties similar to the control material and enhanced in vitro antimicrobial efficiency.

## 1. Introduction

Worldwide, around 2.4 billion people (33% of the population) suffer from dental caries in permanent teeth, and the percentage of this chronic disease increased between 2005 and 2015 by 14% [1]. Moreover, in some countries like Poland, more than 90% of the adult population has experienced dental caries and use dental fillings or dentures [2]. These facts illustrate the progressive extent of the demand for dental materials and the role of constant development in this specific field of material science. Dental caries, but sometimes also dental trauma or extensive wear caused, e.g., by bruxism, may lead to the loss of hard tissues of the teeth. One of the strategies allowing reconstruction of the teeth structure is using direct restorative materials, which are shaped intraorally to create restorations directly in teeth cavities [3]. Currently, the most common of them are photopolymerizable resin-based composites, introduced few decades ago as a substitute for amalgams [4]. This type of material is also considered to be the most prospective, which has resulted in a growing number of new products on the market and numerous investigations in this area. In comparison with other direct restorative materials, composites show optimal esthetic properties, which are related to possibilities of color matching (translucency, shades), satisfying color stability and polishability [5]. Composites are also reasonably easy to use and need less invasive preparation techniques than amalgams [6], which should be considered as additional clinical advantages. As a result of many years of evolution, modern composites show good mechanical and physical properties [7], with wear rates similar to human enamel [8] as well as suitable biocompatibility [9]. Nevertheless, use of resin composites may still lead to higher failure rates in comparison to amalgams [10,11]. The two most frequent reasons for composite failures are fractures and secondary caries [12,13]. Pereira-Cenci et al. [14], in their extensive review, concluded that secondary caries is the cause of up to 55% of resin composite filling replacements. It is defined as “positively diagnosed carious lesion, which occurs at the margins of an existing restoration” [15]. However, currently, it is commonly accepted that it is a primary carious lesion of teeth at the margin of a filling, but it occurs after some time from placing the restoration [15,16], in contrast to the remaining caries, which are caused by incomplete elimination of infected tooth tissues during cavity preparation [15]. Secondary caries is often linked to the presence of microleakage caused by various factors [17,18,19], which may be the reason for the occurrence of liquids, chemical substances, and finally bacteria between the tooth and the restoration [20,21]. Regardless of the doubts about the etiology of caries after the placement of fillings, it is recognized as a serious and widespread clinical problem. Moreover, composites accumulate more biofilm and plaque than other direct restorative materials [22]. For this reason, it is believed that the perfect resin composite filling should not only have suitable mechanical and esthetic properties but also ought to possess antibacterial properties to avoid colonization of the tooth/restoration interface by pathogenic bacteria, such as *Streptococcus mutans* (*S. mutans*) [23,24]. 

Diverse research with different additives has been carried out to develop effective antibacterial composites. Numerous experiments have focused on resins containing polymerizable antibacterial additives, such as quaternary ammonium dimethacrylate (QADM) [25], 12-methacryloyloxydodecylpyridinium bromide (MDPB) [26], dimethylaminohexadecyl methacrylate (DMAHDM) [27], dimethyl-hexadecyl-methacryloxyethyl-ammonium iodide (DHMAI) [28], or dimethylaminododecyl and dimethylaminohexadecyl methacrylates [29]. Other organic materials including quaternary ammonium polyethylenimine (PEI) nanoparticles [30], chlorhexidine [31,32], triclosan [33], chitosan [34], and benzalkonium chloride and acrylic acid [35] were also tested with varying degrees of success. The use of different experimental fillers is another important strategy for developing antimicrobial composites. Tavassoli Hojati et al. [36], Kasraei et al. [37], and Aydin Sevinç et al. [38] reported the reduction of cariogenic bacteria after incorporation of zinc oxide nanoparticles, probably due to the mechanism of production of active oxygen species, such as H_2_O_2_ or the possible leaching of Zn^2+^ ions. Khvostenko et al. [39] used bioactive glass (65% SiO_2_, 31% CaO, 4% P_2_O_5_) and obtained a 61% reduction of *S. mutans* penetration of the gap depth under laboratory conditions, which suggests that the release of ions from glass into the gap may help control the local chemistry by creating an antimicrobial environment that reduces biofilm propagation. Łukomska-Szymańska et al. [20] noted that composites additionally filled with calcium fluoride had shown a significant reduction of *S. mutans* and *L. acidophilus*, which was probably related with creating hydrofluoric acid that can penetrate the bacterial membrane, generate acidification of cytoplasm, and inhibit enzymes. Sodagar et al. [40] modified the commercially available orthodontic composite with titanium dioxide nanoparticles and proved inhibition of *S. mutans* and *S. sanguinis* growth. The most widely tested materials in previous years were those containing silver. Niu et al. [41] successfully applied tetrapod-like zinc oxide whiskers to increase antibacterial resistance. Chatzistavrou et al. [42] confirmed a significant reduction of *S. mutans* for Ag-doped bioactive glass and additional bioactivity of tested materials. Ai et al. [43] investigated composite resin reinforced with silver nanoparticle-laden hydroxyapatite nanowires, where nanowires were used as reinforcement and nanosilver as an antimicrobial agent. The reduction of microorganisms was noted, however, only when the experimental filler was added into the matrix and its concentration was limited to 10%, so those interesting results needs confirmation in follow-up experiments on materials with typical reinforcing fillers. Łukomska-Szymańska et al. [44], reported a viability of *S. mutans* from 48% to 87% in comparison to control samples on the surface of experimental composites with the addition of silver particles alone and combined titanium dioxide, silica dioxide, and zirconium dioxide nanoparticles or microparticles. Kasraei et al. [37] and Azarsina et al. [45] modified commercially available composites with silver nanoparticles and noted a reduction of bacterial colonies. However, amber to brown discoloration of materials with nanosilver has been noted, which is a limitation for an esthetic material [37,44,45]. Also, the inhibitory effect against *S. mutans* of resin composites with silver-containing inorganic particles like silica gel have been confirmed, which the authors linked not with silver ion release but with the presence of active oxygen, including hydroxyl radicals, created by the catalytic action of silver during photoactivation or contact with water at polar surfaces [46]. Additionally, simultaneous effects of silver nanoparticles with hydroxyapatite nanoparticles [47] or antimicrobial monomers [27,48] were also investigated.

Silver sodium hydrogen zirconium phosphate (SSHZP) is a silver-releasing ceramic. This submicron-sized antimicrobial material is white and stable, so as opposed to silver nanoparticles, it should not cause the typical initial amber or brown discoloration due to the plasmon effect [49], which is problematic in the case of dental materials. However, the question of further color changes related with silver ion release during contact with a wet environment and its oxidation remains open. 

So far, SSHZP has been reported as an additive into a polymethyl methacrylate (PMMA) denture base material [50] and a polydimethylsiloxane-based soft denture lining [51]. SSHZP was also previously investigated as an antimicrobial additive into chitosan and alginate fibers [52,53]. Moreover, it is incorporated into some currently available alginate and carboxymethylcellulose wound dressings [54,55]. In this study, we report the use of SSHZP as antibacterial filler for a distinctly different material—a experimental direct restorative photopolymerizable resin-based composite, reinforced with varied filler types at high concentrations. Therefore, the aim of the presented work was to investigate the impact of the proposed filler (SSHZP), introduced into resin-based composites intended as direct restorative materials, for its antimicrobial effectiveness, mechanical properties, degree of conversion, sorption, solubility, and color changes. Our hypothesis was that composites additionally filled with SSHZP would show antimicrobial effectiveness against cariogenic bacteria and suitable properties for dental restorative materials.

## 2. Materials and Methods 

### 2.1. Materials Preparation

The matrix consisted of three mixed monomers: bisphenol A glycidyl methacrylate (bis-GMA), urethane-dimethacrylate (UDMA), and triethylene glycol dimethacrylate (TEGDMA) at a weight ratio of 42:38:20, respectively (all purchased form Sigma-Aldrich, St. Louis, MO, USA). Additionally, 0.4% (*w*/*w*) of camphorquinone (CQ, Sigma-Aldrich, St. Louis, MO, USA) as the photosensitizer and 1% (*w*/*w*) of *N*,*N*-dimethylaminoethyl methacrylate (DMAEMA) as a photoaccelerator (both Sigma-Aldrich, St. Louis, MO, USA) were introduced. The reinforcing fillers were two silanized barium borosilicate glass fillers (Esschem, Linwood, PA, USA), with a mean particle size declared by the manufacturer of 2 μm (G1) or 0.7 µm (G2), and silanized silica nanofiller Aerosil R7200 (AR) (Evonic Industries, Essen, Germany), used at a weight ratio of 50:35:15, respectively. Silver sodium hydrogen zirconium phosphate containing approximately 10% of silver (*w*/*w*), with molecular formula Ag_0.46_ Na_0.29_ H_0.25_ Zr_2_ (PO_4_)_3_ [56] (Milliken Chemical, Spartanburg, SC, USA) was used as an antimicrobial filler. The SSHZP was compounded at concentrations of 1%, 4%, 7%, 10%, 13%, and 16% (*w*/*w*), and the masses necessary to prepare the composites were calculated according to the equation:

(1) $$m_{\mathrm{SSHZP}}\;=\;\frac{c_{SSHZP\;}\times \;m_{MRF}}{1-\;c_{SSHZP}}$$

where m_SSHZP_ was the SSZHP g; *c_SSHZP_* was the SSZHP concentration, % (*w*/*w*); and *m_MRF_* was the matrix with reinforcing filler mass (always constant).

The fillers were compounded into a matrix in 50 mL glass Griffin form beakers at room temperature in the following order: SSHZP, G1, G2, and AR as the last one. All composites were prepared in standardized portions based on the same masses of matrix and reinforcing fillers. The compositions of standardized portions of tested materials are listed in Table 1. The introducing process was carried out gradually in standard portions of 1 g (SSHZP, G1, G2) or 0.5 g (AR). For the lowest concentration of SSHZP, or when the last portion of particular fillers was added, they were smaller. Compounding was effected by multiple spreadings and mixings of materials with a stainless steel spatula on the wall of the beaker to apply shear forces. The subsequent doses of fillers were added when a homogeneous consistency for the previous dose was achieved. The process of compounding for one material took about 2.5–4 h; the longer time was needed for materials with higher filler concentrations due to their increasing viscosity. The obtained compositions were placed under the pressure of 80 mbar for 25 min in a modified vacuum stirrer (Twister evolution, Renfert GmbH, Hilzingen, Germany). All materials were polymerized with a DY400-4 LED lamp (Denjoy Dental, Changsha, China), power 5 W, intensity 1400–2000 mW/cm^2^, optical wave length 450–470 nm.

### 2.2. Scanning Electron Microscopy (SEM) Investigations

Fillers were added to 99.8% ethanol, ultrasonically homogenized, and dropped on carbon tape. Polymerized samples for composite morphology observations measured 10 × 2 × 2 mm. Two types of specimens were used. The first type was subjected to the standard procedure which involved wet-grinding and polishing using diamond pastes. The other type was immersed in liquid nitrogen and broken. Composite samples after polishing were also etched with orthophosphoric acid. All samples were sputtered with gold. Observations were performed using a Zeiss SUPRA 35 scanning electron microscope (Zeiss, Oberkochen, Germany) at accelerating voltages from 3 kV to 20 kV. 

### 2.3. Antibacterial Test

Specimens measured 11 mm in diameter and 2 mm in thickness and were prepared in Teflon molds. The mold was placed at a microscope slide covered with 50 µm thick polyester foil. The material was placed into the mold and covered with the foil and microscope slide. Then, the upper microscope slide was manually pressed and taken away. When the sample was polymerized, the polyester foil was removed. The molds with samples were wet-ground sequentially with P800- and P1200-grit abrasive papers to remove excess of material and to standardize the surface. Next, the samples were rinsed with distilled water and pushed out of the molds. 

The in vitro reduction of bacteria was examined according to the previously described method [51,57,58] with some modifications. The standard strain of bacterium *Streptococcus mutans* ATCC 33535 was used. Sterilized samples of composites were immersed individually in 2 mL of bacterial suspensions in tryptone water, which contained approximately 1.5 × 10^5^ CFU/mL (CFU—colony forming units) of *S. mutans*. A suspension of bacteria in tryptone water was tested as a positive control. Pure tryptone water was tested as a negative control. Incubation was carried out in a shaking incubator for 17 h at 37 °C. After incubation, 20 μL of suspension was seeded onto Columbia agar (bioMerieux, Marcy l’Etoille, France) with 5% sheep blood plates. The cultured plates were finally incubated at 37 °C for 24 h, and the numbers of bacterial colonies were counted. The relative reduction in the number of viable bacteria colonies (RB) was calculated according to the equation:

(2) $$\mathrm{RB}\;=\;\frac{V_{c}-V_{t}}{V_{c}}\times 100\%$$

where V_c_ was the number of viable microorganism colonies of the positive control (BLANK) and V_t_ was the number of viable microorganism colonies of the test specimen. 

### 2.4. Compressive Strength 

Compressive strength was examined according to the method presented by Mota et al. [59], with some necessary specifications concerning sample preparation. Cylindrical specimens (3 mm in diameter and 6 mm in height) were prepared as described for the microbiological test. However, due to their height, polymerization was carried out at the top and at the bottom before the removal of the polyester foil. Furthermore, after removing them from the mold, the samples were cured on four lateral surfaces, according to the recommendation of Galvão et al. [60]. Ten samples were prepared from each composite. The samples were conditioned in distilled water at 37 ± 1 °C for 24 h. Tests were conducted using a universal testing machine (Zwick Z020, Zwick GmbH & Com, Ulm, Germany) at a cross-head speed of 0.5 mm/min. Compressive strength was calculated according to the equation:

(3) $$\sigma_{cs}\;=\;\frac{F}{A}$$

where *σ_cs_* was the compressive strength, MPa; *F* was force at fracture, N; and *A* was the initial cross-sectional area of specimen, mm^2^.

### 2.5. Diametral Tensile Strength

The samples for the diametral tensile strength (DTS) tests (6 mm in diameter and 3 mm in height) [61] were prepared with a method similar to the microbiological test, but irradiation was carried out at the top and at the bottom before removing the polyester foil. Ten samples were prepared from each composite. The samples were conditioned in distilled water at 37 ± 1 °C for 24 h [61]. Compressive load was applied on the lateral surface of the samples at a cross-head speed of 0.5 mm/min [20] using a universal testing machine Zwick Z2.5. The DTS values were calculated according to the equation:

(4) $$\mathrm{DTS}\;=\;\frac{2F}{\pi \mathrm{dh}}$$

where DTS was the ultimate diametral tensile strength, MPa; *F* was the force at fracture, N; d was the diameter, mm; and h was the thickness, mm.

### 2.6. Flexural Strength

Three-point bending tests were carried out using a universal testing machine Zwick Z2.5 in accordance with the ISO 4049 standard [62], with specifications concerning sample preparation. Specimens measuring 25 × 2 × 2 mm were prepared using silicone (Zetalabor Platinum 85Touch, Zhrmack SpA, Badia Polesine, Italy) molds placed in a stainless-steel frame. Materials were packed into a mold and polymerized by a method similar to the previous test, but five overlapping irradiations were carried out, starting from the center of the sample. After curing, samples were taken out of the mold, the excess of material was cut off with a scalpel, and the specimens were then wet-ground with P800- and P1200-grit abrasive papers. Ten samples were prepared from each composite. The samples were stored in distilled water at 37 ± 1 °C for 24 h. The test was performed at a cross-head speed of 0.75 mm/min and the distance between the supports was 20 mm. Flexural strength and flexural modulus were calculated according to the equations:

(5) $$\sigma_{fl}\;=\;\frac{3Pl}{2bh^{2}}$$

(6) $$E\;=\;\frac{P_{1}l^{3}}{4bh^{3}\delta }$$

where *σ_fl_* was flexural strength, MPa; *E* was flexural modulus, GPa; *l* was distance between the supports, mm; *b* and *h* were the specimen width and height, mm; *P* was maximal force, N; *P*_1_ was the load at chosen point at the elastic region of the stress-strain plot, kN; and *δ* was the deflection at *P*_1_, mm.

### 2.7. Vickers Hardness

Vickers microhardness was measured on specimens like for DTS, however, samples after wet-grinding were also polished with 6-µm and 3-µm diamond suspensions (Struers GmbH, Willich, Germany). Three samples were made from each composite. The samples were stored in distilled water at 37 ± 1 °C for 24 h. Hardness was measured 10 times for each specimen at randomly chosen locations using the microhardness tester (Future-Tech FM-700, Future-Tech Corp, Tokyo, Japan) at a 100-g load and a loading time of 15 s [63]. Vickers hardness was calculated according to the equation:

(7) $$E\;=\;\frac{1.8544\times F}{d^{2}}$$

where *F* was the load, N, and *d* was the average length of the diagonal left by the indenter, mm.

### 2.8. Degree of Conversion

The degree of conversion (DC) was determined using the method described by Atira et al. [64] with modifications made during sample preparation. Specimens, measuring 5 mm in diameter and 2 mm in height, were prepared in Teflon molds as previously described, but irradiation was carried out only at the top. The samples were removed from the molds and dried in desiccators with freshly dried silica gel at 37 ± 1 °C for 24 h. Spectra were recorded by a Fourier transform infrared spectroscopy (FTIR) spectrophotometer (Perkin Elmer Spectrum Two, Perkin Elmer, Waltham, MA, USA), equipped with an attenuated total reflectance (ATR) crystal. The absorption intensity of selected peaks was measured in the range of 1800–1500 cm^−1^ and recorded with 128 scans at a resolution of 1 cm^−1^. The DC was calculated from the decrease of the absorption band at 1637 cm^−1^, referring to the C=C stretching vibration (*A_C=C_*) in relation to the peak at 1608 cm^−1^, and assigned to the aromatic stretching vibrations (*A_Ar_*) in accordance with the equation [65]:

(8) $$\mathrm{DC}\left(\%\right)\;=\;\left(1-\frac{{\left(A_{C=C}/A_{Ar}\right)}_{after\;curing}}{{\left(A_{C=C}/A_{Ar}\right)}_{before\;curing}}\right)\times 100$$

### 2.9. Sorption and Solubility

The specimens measuring 15 mm in diameter and 1 mm in height were prepared using Teflon molds [66] and polymerized at nine overlapping irradiation zones in accordance with the method described in the ISO standard [62]. After curing, they were ground with P1200-grit abrasive paper to remove excess material with potentially poorly polymerized layers [67] and to standardize the surface. Then, the samples were removed from the molds. Five test samples of each material were made. The measurement of sorption and solubility was performed in accordance with ISO 4049. The samples were dried inside desiccators with freshly dried silica gel in a dryer at 37 ± 1 °C and weighed daily (AS 110/C/2, Radwag, Radom, Poland) with an accuracy of 0.1 mg. When the changes in mass were no higher than 0.1 mg, the mass values were recorded as m_1_, and the thickness and diameter were measured with a digital caliper with an accuracy of 0.1 mm. Each sample was placed in 10 mL of distilled water for 7 days at 37 ± 1 °C. After storing, the samples were removed from water with tweezers, dried from visible moisture with filter paper, kept at room temperature for 15 s, and weighed (m_2_ mass values were denoted). The drying process was repeated as described above, and stable mass was denoted as m_3_. Sorption and solubility were calculated using equations:

(9) $$w_{\mathrm{sp}}=\frac{m_{2}-m_{3}}{V}$$

(10) $$w_{\mathrm{sl}}=\frac{m_{1}-m_{3}}{V}$$

where w_sp_ was sorption, w_sl_ was solubility, m_l_ was the initial mass of dried sample, µg; m_2_ was the mass after storing, µg, and m_3_ was the mass after the second drying, µg; and V was the volume of the sample, mm^3^.

### 2.10. Color Change Measurement

To evaluate the color changes, the specimens measuring 7 mm in diameter and 3 mm in thickness were prepared in Teflon molds. The mold was placed on a microscope slide. The material was placed into the mold, covered with polyester foil and finally with second microscope slide. Then, the upper microscope slide was manually pressed and taken away. The form prepared in this way was inverted (the slide was on top, foil on the bottom). This was important to do because during polymerization, the elastic foil allowed the material to move due to polymerization shrinkage (typical meniscus was formed), while the working surface of the composite in contact with the slide adhered to it and remained flat. The cured sample was pushed out of the mold. Five samples were prepared from each material. After preparation, samples were stored in dry and dark conditions at 37 °C for 24 h and next were immersed in 10 mL of distilled water in darkness at 37 ± 1 °C. Distilled water was replaced after the second and fourth day. Color measurements were obtained 24 h after polymerization (baseline) and after 7 days of immersion. A spectrophotometer (CM2600d, Konica Minolta, Takyo, Japan) was used to record the CIE L*a*b* parameters with a D65 illuminant on a white ceramic tile. The CIELab system is composed of three axes: L* is the lightness from 0 (black) to 100 (white), a* represents the red (+a* value)—green (−a* value) axis, and b* represents the blue (−b* value)—yellow (+b* value) axis. The color change (ΔE*) was calculated using the equation [68]:

(11) $$\Delta E*=\sqrt{{\left(\Delta L*\right)}^{2}+{\left(\Delta a*\right)}^{2}+{\left(\Delta b*\right)}^{2}}$$

where ΔL* = L_(7 days)_ − L_(baseline)_; Δa* = *a*_(7 days)_ − *a*_(baseline)_; and Δb* = *b*_(7 days)_ − *b*_(baseline)_.

### 2.11. Statistical Analysis

Statistical analysis of the results was done with the use of the Statistica software (software version 13.1, TIBCO Software Inc., Palo Alto, CA, USA). The distributions of the residuals were tested with the Shapiro–Wilk test, and the equality of variances was tested with the Levene test. When the distribution of the residuals was normal and the variances were equal, the one-way or two-way ANOVA with Tukey HSD post hoc tests were used (α = 0.05), otherwise the nonparametric Kruskal–Wallis test (α = 0.05) was used. Regression analysis was performed to determine the correlation between DC and hardness (α = 0.05).

## 3. Results

### 3.1. Scanning Electron Microscopy Investigations

**Figure 1:**
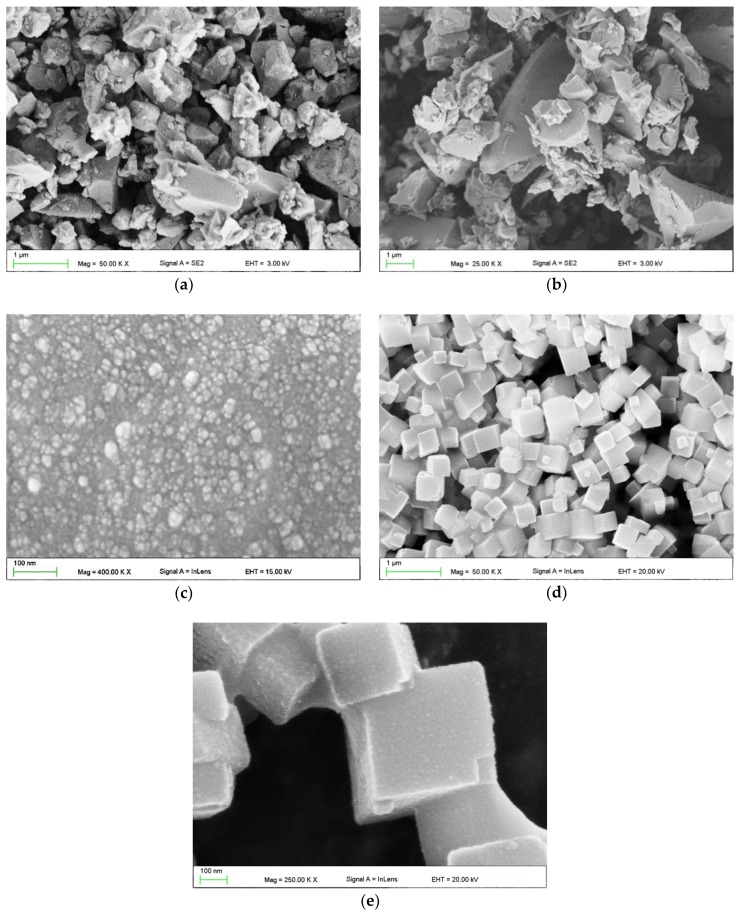
Scanning electron microscopy images presenting the morphologies of used fillers: glass fillers with a mean particle size of 0.7 μm (**a**); 2 μm (**b**); silica nanofiller (**c**); and silver sodium hydrogen zirconium phosphate (**d**,**e**).

Figure 1 presents the morphologies of the used fillers. For both glass fillers (Figure 1a,b), numerous particles showed a much smaller (starting from 50 nm) or larger (up to 8 µm) size than the mean size declared by the manufacturer (2 µm and 0.7 µm). The shapes of the particles were irregular. Nanoparticle aggregations measuring up to 50 nm were noted for silica filler (Figure 1c). For SSHZP particles measured approximately from 100 nm to 500 nm (Figure 1d) but also larger structures, consisting of particles connected to each other, were observed (Figure 1e). 

**Figure 2:**
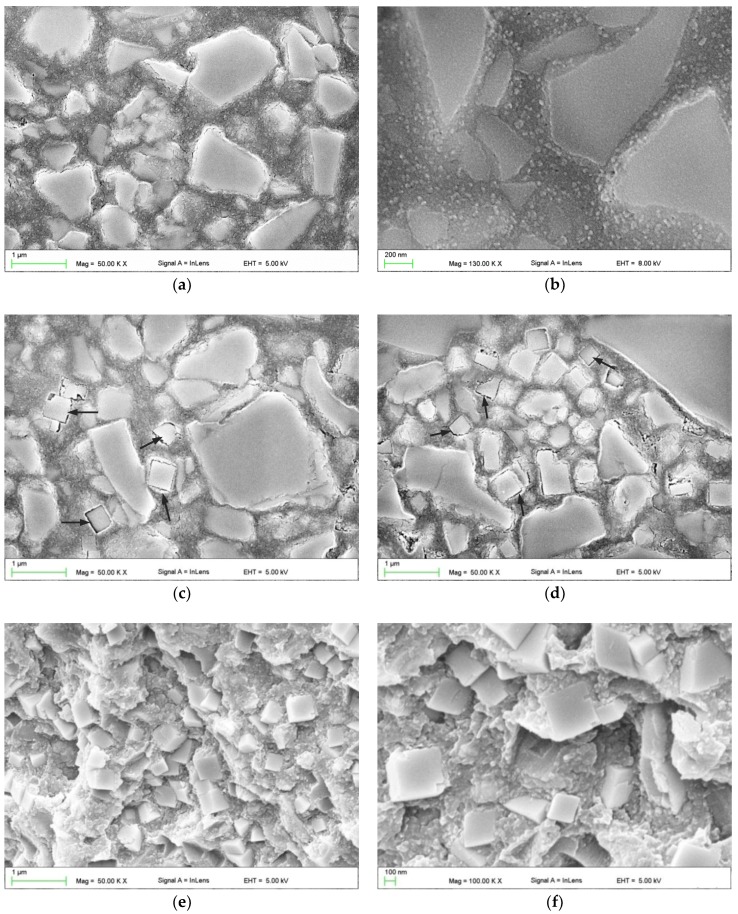
Representative SEM images presenting the morphologies of the cured base composite compounded with: reinforcing fillers (**a**,**b**); addition of 7% (**c**) and 16% (**c**–**f**) of silver sodium hydrogen zirconium phosphate; (**a**–**d**)—wet-ground, polished, etched samples (**e**,**f**)—frozen-broken but not etched samples, black arrows (**c**,**d**) indicate the gaps between SSZHP and matrix after etching.

SEM images illustrating the morphologies of composite reinforced with glass and silica fillers are presented in Figure 2a,b. The morphologies of materials with additional antibacterial filler are presented in Figure 2c–f. Good distribution of silica nanoparticles between glass submicroparticles and microparticles in the matrix was observed (Figure 2b). Large aggregations of AR were not detected. The SSHZP was also well distributed up to the highest concentrations. Single particles were clearly visible, however, clusters measuring up to 2 µm were also noted. Observations for frozen-broken but not etched samples (Figure 2e,f) showed good contact between the particles and the matrix.

### 3.2. Antibacterial Test

The achieved results of the antibacterial tests are listed in Table 2. Introducing the SSZHP into the composites had a significant effect (*p* = 0.0002) on the reduction of *S. mutans* colonies. For material without antimicrobial filler, RB values were comparable to the positive control. Composites with filler concentrations from 1% to 4% showed RB medians from 43.8% to 70.1%, and those values should be considered as different if we take into account the obtained minimal and maximal RB values. For concentrations starting from 7%, all obtained RB values were 100%.

### 3.3. Compressive Strength 

**Figure 3:**
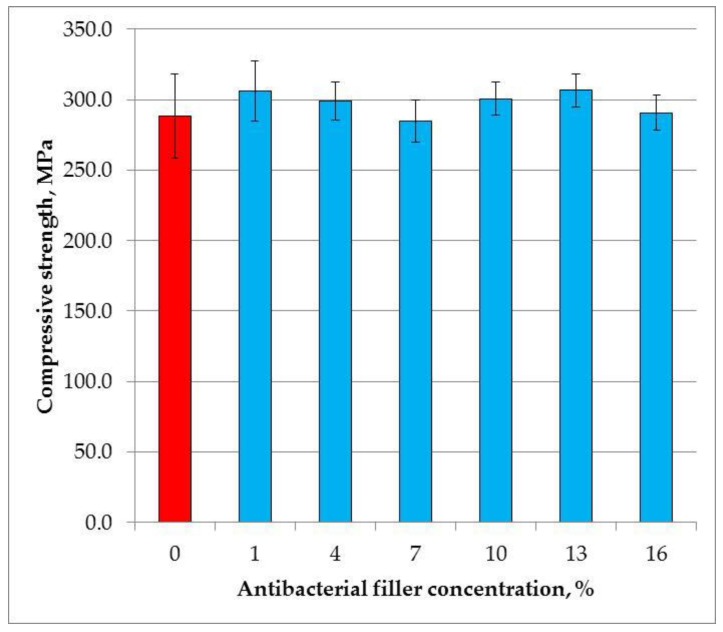
Mean values and standard deviations of compressive strength.

The mean compressive strength values are presented in Figure 3. The SSHZP concentration did not have a significant influence on the compressive strength of the composites (*p* = 0.0524). The mean values were from 284 MPa to 307 MPa.

### 3.4. Diametral Tensile Strength

**Figure 4:**
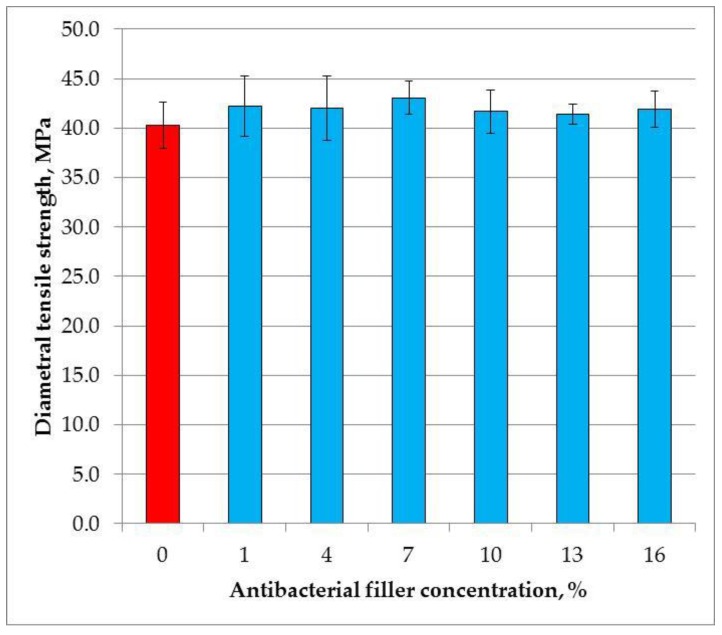
Mean values and standard deviations of diametral tensile strength.

The mean diametral tensile strength values are presented in Figure 4. The SSHZP concentration did not have a significant influence on the compressive strength of the composites (*p* = 0.2986). The mean values were from 40.3 MPa to 43.1 MPa.

### 3.5. Flexural Strength

**Figure 5:**
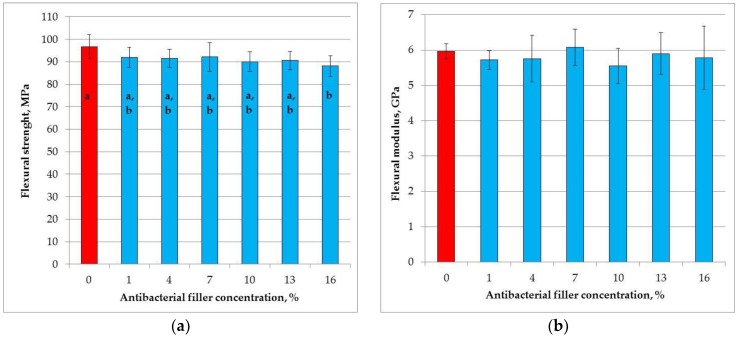
Mean flexural strength (**a**) and flexural modulus (**b**) values with standard deviations; different lowercase letters show significantly different results at the *p* < 0.05 level.

The mean flexural strength values are presented in Figure 5a. The SSHZP introduction had a significant influence on flexural strength (*p* = 0.0178). The post hoc test showed a significant (*p* < 0.05) decrease in flexural strength for the composite with the antibacterial filler concentration of 16% (88 MPa). However, these values were not significantly different (*p* > 0.05) in comparison to the results obtained for other materials with SSHZP. The highest mean flexural strength value was registered for the control material (96 MPa). 

The mean flexural modulus values are presented in Figure 5b. The SSHZP concentration did not have a significant effect on the flexural modulus (*p* = 0.5351). The mean values were from 5.6 GPa to 6.1 GPa. 

### 3.6. Vickers Hardness

**Figure 6:**
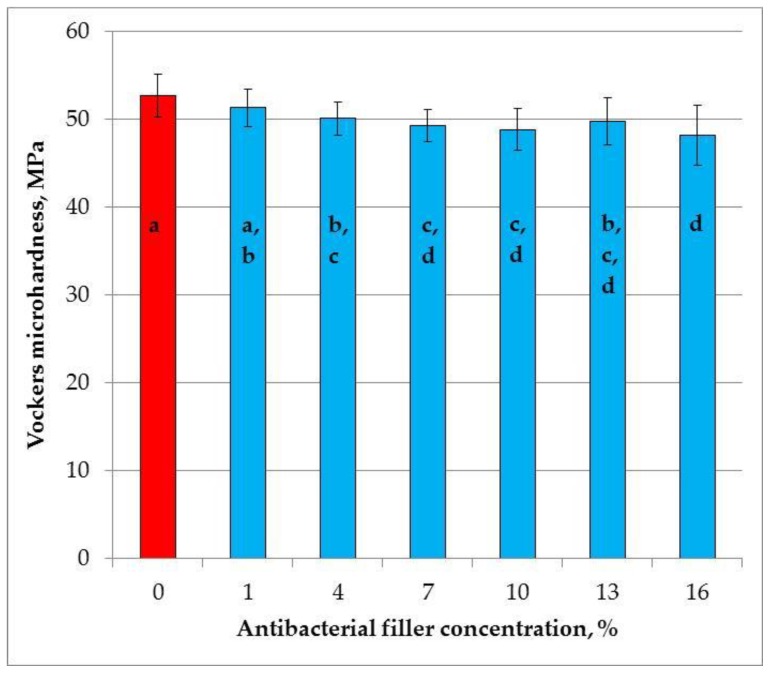
Mean Vickers microhardness values with standard deviations; different lowercase letters show significantly different results at the *p* < 0.05 level.

The mean Vickers hardness values are presented in Figure 6. The SSHZP introduction had a significant influence on flexural strength (*p* < 0.0001), and the post hoc test showed a significant (*p* < 0.05) decrease in hardness starting from the antibacterial filler concentration of 4%. However, the values for SSHZP concentration from 4% to 13% and from 7% to 16% were not significantly different. The highest mean hardness value was registered for the control material (52.7 HV0.1), and the lowest value was for a composite with 16% of SSHZP (48.2 HV0.1). 

### 3.7. Degree of Conversion

**Figure 7:**
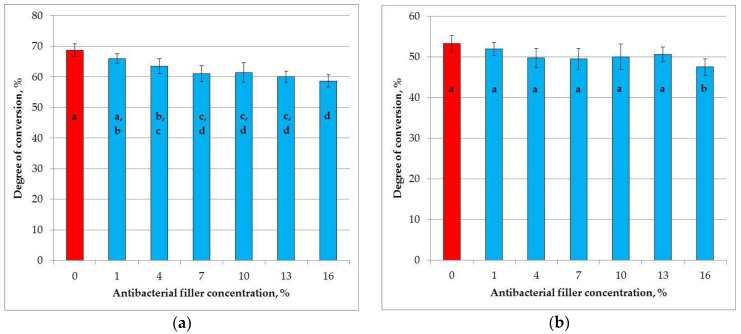
Mean degree of conversion values with standard deviations at the top (**a**); and at the bottom (**b**) of the samples, different lowercase letters show significantly different results at the *p* < 0.05 level.

The mean degrees of conversion values are presented in Figure 7. At the top of the samples (Figure 7a) the degree of conversion significantly decreased (*p* < 0.0001) with increasing SSHZP concentrations, from 68.7% for the control material to 58.7% for the composite with an SSHZP concentration of 16%. The post hoc test showed that the results for concentrations from 1% to 7%, from 4% to 13%, and from 7% to 16% were not significantly different. Degrees of conversion values obtained at the top were significantly lower in comparison to the values registered at the bottom of the samples (*p* < 0.0001). At the bottom of the samples (Figure 7b), the degree of conversion significantly decreased (*p* < 0.0134) with increasing SSHZP concentrations, from 53.3% for the control material to 47.6% for the composite with an SSHZP concentration of 16%. However, the post hoc test showed that the results for concentrations from 1% to 13% were not significantly different, and only the mean value for the material with the highest SSHZP concentration was significantly lower. 

### 3.8. Sorption and Solubility

**Figure 8:**
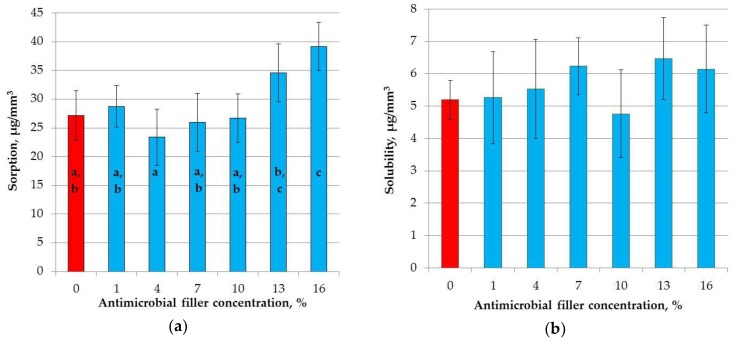
Mean values with standard deviations of sorption (**a**); and solubility (**b**), different lowercase letters show significantly different results at the *p* < 0.05 level.

The mean sorption values are presented in Figure 8a. SSHZP introduction had a significant influence on sorption values (*p* < 0.0004). The post hoc test showed a significant increase in sorption values for composites with 13% and 16% of SSHZP. The mean sorption for the composite with the highest SSHZP concentration was 44% greater than for the control material.

The mean solubility values are presented in Figure 8b, and there were no statistically significant differences (*p* = 0.4185) between the results obtained for the investigated materials.

### 3.9. Color Measurement

The results of initial color measurements are presented in Table 3. The obtained L* axis values showed a significant increase (*p* < 0.0001) with the increasing SSHZP concentration. The a* and b* axis values showed a significant decrease with the increasing SSHZP concentration (*p* < 0.0001).

The color changes of different composites after immersion in distilled water are presented in Table 4. The ΔE values for the different composites showed a significant increase (*p* < 0.0001) with the increasing SSHZP concentration. However, the post hoc test indicated significant differences in comparison to reference materials for composites with 13% and 16% of SSHZP. A similar situation was registered for ΔL* values. The statistically significant influence (*p* < 0.0001) of SSHZP concentration was also noted for Δa* and Δb* values. The post hoc test showed a significant increase for composites with 13% and 16% of antibacterial filler. 

## 4. Discussion

In the current study, experimental composites based on a photopolymerizable matrix were considered as direct antibacterial restorative materials. Materials were developed by introducing a filler with confirmed antimicrobial properties: silver sodium hydrogen zirconium phosphate particles. In previous experiments, we tested SSHZP as an antimicrobial additive in two different dental materials: a PMMA denture base material [50] and silicone soft denture lining [51]. Both types of composites had shown enhanced antimicrobial properties, but for the PMMA-based materials, a significant deterioration of mechanical properties had been registered (results unpublished yet), while for polydimethylsiloxane-based composites, they were at the appropriate level [51]. In the presented work, we modified different materials in terms of the final application, polymerization, mechanical properties, and composition. 

The applied filler compounding method allowed us to obtain satisfactory dispersion of the used fillers. Observations carried out on polished and etched samples clearly showed typical, irregular shapes of milled glass particles and a very good distribution of nanoparticles between them (Figure 2b). Aggregations of glass fillers or large AR aggregations were not detected. When SSHZP was additionally introduced, cubic-shaped particles were well distributed between glass particles. Due to the used filler types and the obtained morphology, all used materials may be classified as nanohybrid composites [69]. Gaps were visible between the matrix and SSHZP particles (Figure 2c,d), which was related to etching during sample preparation. For nonetched samples, slits were not detected with the used method (Figure 2e,f). However, the observed gaps may suggest the possibility of easier liquid migration between SSHZP and the matrix than between silanized glass and the matrix. For frozen-broken nonetched samples, glass particles were usually not visible, which suggests their good connection with the matrix and is related to the salinization process used by the manufacturer. Large aggregations of SSHZP were not observed, which was favored because of their potential influence on the properties of the composites [70]. However, some structures consisting of connected particles, probably coming from the used antimicrobial filler (Figure 1e), were observed. Observations have also shown some air bubbles in the polymerized composites. They were probably caused by the used procedure of manual preparation of composite and/or by the process of sample preparation. Bubbles measured from a few up to 50 µm. Those structural defects might decrease the mechanical properties because they may act as stress concentrators. In the future, the bubbles can also have a negative effect on the mechanical properties at the bonded interface.

In previous works related with dental materials, the antimicrobial effectiveness of SSHZP against *Candida albican* (*C. albicans*), *Staphylococcus aureus* (*S. aureus*), and *Escherichia coli* (*E. coli*) was confirmed [50,51]. However, only two tested microorganisms (*C. albicans*, *S. aureus*) had clinically proven relevance, which is related to using partial or complete dentures [71,72,73,74], but none of them was associated with tooth decay. The problem of caries appearing between teeth and composite restorations is widely disputed in the literature, and its mechanisms are probably multifactorial. Bacterial species associated with secondary caries and primary caries seem to be the same. However, a higher proportion of caries-related bacteria (*mutans streptococci*, *lactobacilli*) was found on restored surfaces than on unrestored dentin or enamel [75], which is an additional argument for the development of antibacterial materials. Despite the fact that both *mutans streptococci* and *lactobacilli* have a confirmed role in dental caries, the *S. mutans* strains are usually investigated in the context of antimicrobial composites, so this bacterium was also used in our experiment.

Due to differences in microbiological test protocols, the results obtained for the antimicrobial fillers mentioned in the introduction cannot be directly compared to one another or to the results of the present study. In our experiment, samples were stored in an *S. mutans* suspension. All specimens were finally finished with P1200-grit abrasive paper, which gives well standardized and smooth surfaces. This may be confusing in the context of the recognized fact that higher values of roughness promote bacterial adhesion and dental plaque retention [76,77,78]. However, in our study, adherence of bacteria and biofilm formation were not investigated. Samples were immersed in bacteria suspension in a shaking incubator, and the changes in the number of bacteria were investigated. In this experiment, silver ions released into the environment determined the reduction of bacteria, so using a smooth surface would have created stricter test conditions due to the smaller surface area responsible for the release of antibacterial ions. 

After incubation, the reduction of the bacteria population in the environment was registered for all materials, but starting from a concentration of 7%, it was complete. Antimicrobial properties of the used filler are initiated in humid environments by the mechanism of silver ion release from an inorganic, insoluble carrier, which was described by Kampmann et al. [79]. With time, this mechanism may lead to the loss of antibacterial properties due to the continuous silver ion release, so further investigations in this context should be made. Additionally, restorative materials in oral cavities are subject to tribological processes. This, on the one hand, may be the reason for the selective removal of particles from the matrix, but on the other hand, it can also cause “refreshment” of antimicrobial properties by gradual abrasion of the surface with fillers. Both mentioned conceptions may be checked in future experiments. 

The microbiological properties of the newly developed antibacterial composites are regularly tested, whereas their mechanical and functional properties are much less frequently reported. Compressive strength, flexural strength, flexural modulus, diametral tensile strength, and hardness are frequently tested mechanical properties for dental restorative materials. 

In the present study, we used different curing protocols for each mechanical evaluation. It was justified by the varied sample dimensions, which were dictated by the requirements of the procedures. It is known that light intensity, polymerization time, and curing depth determine whether or not dental composites are properly cured [80]. Mechanical tests as well as sorption/solubility and antibacterial tests require samples having a length or diameter much larger than the area effectively covered by the used lamp. The use of overlapping light-curing areas for flexural test samples has been subject to criticism due to the risk of preparation of nonhomogeneous specimens [81], although the effect of that method on the flexural properties has been questioned [82]. The height of the samples is equally important due to the expected decrease of the degree of conversion with the depth [83]. A thickness of 2 mm can be considered in that context as a safe value [84]. Moreover, Koran et al. [85] established that if the total dose of light intensity (interpreted as light intensity in exposure time) delivered to the photopolymerizable dental composite is high enough to achieve complete polymerization, the surface hardness, as well residual monomer concentration, tends to remain constant. This shows that the best way to standardize samples is to use overlapping areas of irradiation on both the bottom and top surface, and for samples higher than 4 mm, to use additional irradiations on the lateral surface. However, such an approach is a simplification because it does not take into account other changes occurring in the material during the polymerization and postirradiation polymerization [86,87,88].

Composites often replace a large bulk of the teeth structure, so the dental restorative materials are usually subjected to compressive forces generated during mastication [89]. If we consider that the compressive strength and plastic limit of tooth tissues [3,90] may be recommended as a standard for the strength of composites [60], we can accept a 230 MPa as a secure value for a composite. The obtained results were higher and were additionally comparable with values reported for numerous commercially available materials with BisGMA, TEGDMA, and UDMA matrices and similar filler content [59,91], including those releasing fluoride [92]. Compressive strength after antimicrobial filler addition was investigated in only a few previous works. The effect of the used additives was varied. An increase of compressive strength values at low concentrations and a decrease of them at larger concentrations was noted for nanosilver [93], zinc oxide [36], and tetretrapod-like zinc oxide whisker [41]. Yoshida et al. [74] have shown no effect of silver-containing ceramic microparticles. In our study, the filler addition also had no effect on compressive strength.

Stress analyses have shown that restorative composites can fracture under tension [94] and tensile strength data may have equal, if not greater, importance than compressive strength, especially in the area near the teeth–composite interface [95,96]. The diametral tensile strength test is an alternative method to evaluate the tensile strength of brittle materials, and it is the default for investigating dental restorative materials. Nevertheless, it gives correct results only if minimal or no plastic deformations occur and when deformations at fracture are small because the area of contact is still near to theoretical [61]. For this reason, this test should not be used for resins or experimental composites with a low filler concentration because of their stress-strain characteristics. Usually DTS values for different types of commercially available composite materials range from 25 MPa to 50 MPa [97,98,99], but for modern nanocomposites, they may reach over 80 MPa [100]. For all investigated materials, mean DTS values were above 40 MPa, which can be considered to be satisfactory values. The antibacterial filler introduction had no statistically significant effect on DTS, although the mean value for the control group was the lowest. Those findings are in opposition to the results obtained by Łukomska-Szymańska et al. [101], Diaz et al. [102], and Sokołowski et al. [103], where nonfunctionalized calcium fluoride microparticles, zinc oxide three-dimensional microstructures, nanosilver, and nanogold decreased the DTS values of modified composites. 

Flexural strength and flexural modulus have been reported as indicators of clinical wear of composites in some studies [104,105]. Composite fillings are also exposed to flexural stress, especially in stress-bearing cavities for restoration classes I, II, and IV [82]. The flexural test is also indicated as a method that relates well to tensile failure [104]. Flexural strength is the only mechanical property specified by the ISO 4049 standard for composite restorative materials, which requires minimal values of 80 MPa for occlusal tooth surface restorations and 50 MPa for others [62], so all investigated materials meet these requirements. The obtained results were additionally comparable to other materials with similar matrix or filler concentrations [4,106,107,108]. A parallel situation was noted for flexural moduli [109], the values of which are not defined by the standard and may be diversified for different clinical situations. Cervical cavities demand composites characterized by a relatively lower modulus to flex with the teeth, but posterior composites need a high modulus to withstand the occlusal forces [110]. In the presented study, the flexural strength significantly decreased only for the highest antimicrobial filler concentration, whereas the flexural modulus values were stable. Similar trends were noted for other experimental materials compounded with antibacterial particles [36,111]. However, for composites with calcium fluoride, after 24 h storing in wet conditions, a significant deterioration of flexural properties has been registered [112]. The addition of zinc oxide whiskers [41] and nanoparticle-laden hydroxyapatite nanowires [36] caused increases in flexural strength and modulus, but for the concentration of 10%, properties decreased for both fillers. This suggests that the obtained effects are related to both filler type and filler loading. The decrease in flexural strength noted in our study for the highest SSHZP concentration should be treated with some caution, also in the context of the obtained compressive and tensile strength values. For one sample, the flexural strength was 77 MPa. If we deleted this result, we would have a mean value of 90.1 MPa, and this value is not statistically different from any other. After consideration, we decided not to remove that result because samples for flexural strength for this material were the most difficult to prepare due the increasing viscosity of composition in combination with a small working area of silicone mold (2 × 25 mm), which created an increased risk of making structural defects during the packing of the material. The registered statistical difference can be an indicator of those problems, especially if we consider that flexural strength has been noted to be more sensitive to subtle changes in material substructure than, for example, compressive strength test [113]. 

The Vickers microhardness test is a known method used to compare composite resins, especially in the context of their wear resistance prediction [50,114]. Direct restorative composites used in dentistry demonstrate Vickers hardness starting from 40 kgf/mm^2^, but for some materials, values exceed 100 kgf/mm^2^ [10,114,115,116]. The values obtained in this work were within this range but were rather at the lower limit. Increasing antibacterial filler concentration caused a small but systematic lowering of microhardness. This is in opposition to some findings for commercially available materials, where higher filler content was correlated with higher microhardness [117], but is in accordance with some research, where introducing antibacterial additives decreased microhardness [44,101,111]. 

Degree of conversion is an important property of restorative composites due to the potential risk of biological responses related to monomer release and affection of pulp tissues [118]. The obtained values were in agreement with the findings from other studies, performed on similar dimethacrylate systems, and measured with the same method [64]. The reduction of DC values at the bottom of the samples was also expected because when light moves through a material with increasing density, its intensity is reduced. The reduction of DC values with increasing SSHZP content was probably related to the effect of light scattering by particles. Moreover, some reports showed larger scattering when the particle size is circa one half or close to that of the curing light wavelength [119,120]. In the presented study, this situation took place because the optical wave length was 450–470 nm, so it was similar to the observed SSHZP particles size, which may explain unfavorable DC changes. Additionally, the DC values at the top of the samples were well correlated (*R*^2^ = 0.9058, *p* = 0.001) with microhardness results. This is in accordance with other reports, where surface microhardness has been identified as a good indicator of DC changes [121,122]. 

The solubility and sorption properties are important from the viewpoint of biocompatibility concerns over monomer releasing and in relation to the stability of the composites due to degradation from the uptake of solvents and the wash-out of ingredients of materials [104]. Sorption values for all samples were lower than 40 μg/mm^3^, so all investigated materials met the requirements of the ISO standard. Solubility values’ samples were lower or, for one sample, equal to 7.5 μg/mm^3^, so all materials also met the requirements of the ISO standard. The sorption values were comparable to numerous commercially available materials [123,124] but increased for the highest antimicrobial filler concentration. The solubility for all composites, including the control group, was generally higher than for most modern materials, however, for some of them, a similar value has also been registered [66,123,124]. The increasing solubility with SSHZP content was not statistically significant. The principal factor influencing sorption and solubility of dental restorative materials is the composition of polymer matrix [67]. The sorption is influenced by the polarity of the molecular structure, the presence of hydroxyl groups (which may crate hydrogen bonds), and with the degree of crosslinking of the matrix [125]. Water may penetrate into the free volume between the chains and nanopores formed during polymerization, or it can be successively attached to polymer chains via hydrogen bonds [123]. In this study, a hydrophilic monomers system (Bis-GMA, TEGDMA, UDMA) [123] was used, from which TEGDMA and Bis-GMA create networks characterized by higher sorption due to the presence of the ether linkages and hydroxyl groups, respectively [66,126]. Thus, those kinds of matrices usually show relatively large values of water sorption and solubility. Additionally, after the introduction of nonsilanized SSHZP, water would migrate in the interface between the filler particles/particle aggregations and the matrix, which may explain the enhanced sorption values for the higher concentration. Also, decreased DC may have some influence on water sorption. The uptake of water also allows diffusion out (into the storage medium) to residual monomers, fillers, degradation products, and other leachable components, so sorption is often correlated with solubility [66]. This was not found in this study, although a statistically insignificant increase in solubility was registered. This may suggest that an experiment should be prepared in future with longer storage periods to allow for more extensive changes in the materials, which will be easier to detect, or with more sensitive methods. 

The introduction of SSHZP caused significant changes in composite color. The materials with increasing antimicrobial filler concentrations show a narrow whitening effect, represented by increasing L* axis values. Composites have also shown a reduction of reddish and yellow coloration related to decreasing positive values of a* and b*, respectively. These changes, at the starting point, might be considered as beneficial. After being stored in distilled water, color changes expressed by ΔE values can be classified as noticeable only for an experienced observer (ΔE values from 1 to 2) for all materials, excluding the highest concentration for which unexperienced observer may notice the difference (ΔE values from 2 to 2.5) [127]. However, all achieved ΔE values were comparable to the results obtained for commercial [128] and experimental [129,130] materials after a similar period of storing in distilled water. For all composites, the darkening, reddening, and yellowing effects were registered, which were also registered for other photopolymerizable resin-based composites [120,128,130]. The reasons for the noted color changes might be multifactorial. De Oliwiera et al. [120] suggest that reduced monomer conversion can lead to poorer color stability in the composites during storing due to the oxidation of the amines or monomers. In our study, the reduction of DC values was also noted, which may partially be the reason for the reddening and yellowing of the materials. However, the slight, progressive changes in DC values cannot explain much of the decreased color stability of the materials for the two highest concentrations of SSHZP. The effect of color changes can be also associated with the leaching of components [130], including the antibacterial filler. The silver ions released from the filler, their deposition on the surface, and further oxidation may be considered as the reason for the darkening, reddening, and yellowing of the composites. However, additional investigations ought to be conducted to clarify this behavior.

The findings presented here should be enhanced with further in vitro investigations. As an especially important part of future research, the cytotoxic potential of composites should be examined. The toxicological data for the used SSHZP [56] let us suppose that materials should not present unfavorable properties in that aspect, although toxic effects in dental materials with silver have been registered [70]. The ion release into the environment and the dynamics of this process should be investigated, as an indication of the persistence of the material’s antibacterial activity. The SSHZP introduced into the PMMA denture base material in a previous work have shown antimicrobial properties decreasing with time during a three-month experiment [50], so it should be expected that the durability of the antibacterial activity of the tested composites will also be limited. The used antimicrobial test additionally did not reflect real conditions, especially in the context of the expected lifetime of the dental composites and the processes occurring in the interface area between tooth tissues and composite. Nevertheless, the presented results of antimicrobial tests are a promising base for further experiments. All properties studied here may also be tested with long-term experiments. 

Another limitation of the present study was that the mechanical properties of the proposed composites were not evaluated after biofilm exposure. Acid production by bacteria during metabolic processes may be the cause of changes in some mechanical properties of composites. Microhardness is usually stable after exposure to *S. mutans* biofilm [77,131], but Fúcio et al. [131] have shown that it can be reduced for particular restorative composites. Moreover, the presence of biofilm influences the mechanical properties of resin–dentin bonds. Melo et al. [132], in self-designed experiments with quasi-static and fatigue performance tests, have shown that the *S. mutans* growth may be the cause of the reduction in the mechanical properties of the bonded interface. The degradation in the dentin–composite interface in the biofilm environment was confirmed by other researchers [133,134], who indicated that the studied experimental composites should be examined in the future in this respect. The problem of acid production by bacteria may also be important in the context of the observed gaps between SSZHP particles and the matrix after the etching of samples for SEM investigations. This may suggest the possibility of accelerated changes in this area due to the influence of the acidic environment. Depending on whether these changes occur as a result of the presence of a biofilm, this phenomenon could negatively affect the mechanical properties of materials due to the creation and propagation of structural defects.

In the present study, we used quasi-static tests for the mechanical properties’ evaluation, which was sufficient at the planned initial stage. However, fatigue tests in the past decade have gained increased importance because dental materials under clinical conditions are subject to cyclic loading [135], caused by thousands of cycles of mastication per day. It has been proven that flexural static strength is higher than values obtained after flexural cyclic loading [136]. Cyclic loading also reduces fracture toughness [137]. Fatigue tests are also used to evaluate mechanical properties of resin–dentin bonds [138,139], also in the context of bacteria presence [133]. These results suggest the desirability of conducting research in this direction, including comparative studies with commercially available composites.

## 5. Conclusions

Within the limits of this study, it can be concluded that the experimental composites showed a high initial reduction of bacteria colonies for the tested *S. mutans* strain. The satisfactory combination of the reduction of bacteria colonies with physical properties was achieved for filler concentrations ranging from 4% to 13%. Those materials exhibited mechanical properties similar to the base material, as well as the degree of conversion, sorption, solubility, and color stability at acceptable levels. The cytotoxic tests and long-term investigations, including silver ion release into the environment, need to be performed in future experiments.

## Figures and Tables

**Table 1:** Compositions of investigated materials with the masses of components needed to prepare standard portions.

Code	Matrix, g	Matrix, % (*w*/*w*)	RF, g	RF, % (*w*/*w*)	SSHZP, g	SSHZP, % (*w*/*w*)	TF, % (*w*/*w*)
Control	15.00	35.00	27.86	65.00	0	0	65.00
AC 1	15.00	34.35	27.86	64.65	0.43	1	65.35
AC 4	15.00	33.60	27.86	62.40	1.76	4	66.40
AC 7	15.00	32.55	27.86	60.45	3.22	7	67.45
AC 10	15.00	31.50	27.86	58.50	4.76	10	68.50
AC 13	15.00	30.45	27.86	56.55	6.40	13	69.55
AC 16	15.00	29.40	27.86	54.60	8.16	16	70.60

AC—antibacterial composite, RF—reinforcing fillers, SSHZP—silver sodium hydrogen zirconium phosphate, TF—total concentration of compounded fillers.

**Table 2:** The reduction in the number of viable colonies (RB) of *Streptococcus mutans* ATCC 33535, after 17 h of incubation with composites samples.

c_SSZHP_, %	CFU/mL (V_t_) ×10^4^			RB, %		
	Med	Max	Min	Med	Max	Min
0	3.53	3.99	3.13	4.7	15.5	−7.7
1	2.08	2.89	1.87	43.8	49.6	21.9
4	0.68	0.13	0.00	70.1	93.2	65.7
7	0.00	0.00	0.00	100.0	100.0	100.0
10	0.00	0.00	0.00	100.0	100.0	100.0
13	0.00	0.00	0.00	100.0	100.0	100.0
16	0.00	0.00	0.00	100.0	100.0	100.0

c_SSZHP_—concentration of silver sodium hydrogen zirconium phosphate; CFU—colony forming units; RB—the relative reduction in the number of viable bacteria colonies; Med—median, Min—minimal value, Max—maximal value.

**Table 3:** The color of different composites before immersion.

c_SSZHP_, %	L*	a*	b*
0	51.34 ± 0.36 ^a^	5.97 ± 0.08 ^a^	12.83 ± 0.26 ^a^
1	54.48 ± 0.47 ^b^	4.53 ± 0.20 ^b^	11.21 ± 0.38 ^b^
4	70.42 ± 0.21 ^c^	2.28 ± 0.21 ^c^	10.59 ± 0.35 ^c^
7	76.50 ± 0.18 ^d^	1.79 ± 0.15 ^d^	9.55 ± 0.22 ^d^
10	81.09 ± 0.09 ^e^	1.00 ± 0.04 ^e^	6.82 ± 0.18 ^e^
13	82.84 ± 0.16 ^f^	0.96 ± 0.07 ^e^	5.63 ± 0.10 ^f^
16	85.84 ± 0.16 ^g^	0.51 ± 0.11 ^f^	5.32 ± 0.19 ^f^

Groups with the same lowercase superscript letters for each column are not significantly different at the *p* < 0.05 level.

**Table 4:** The color changes of different composites after immersion.

c_SSZHP_, %	ΔL*	Δa*	Δb*	ΔE*
0	−0.88 ± 0.15 ^a^	0.37 ± 0.07 ^a^	0.61 ± 0.04 ^a^	1.14 ± 0.10 ^a^
1	−0.95 ± 0.18 ^a,b^	0.41 ± 0.06 ^a^	0.64 ± 0.04 ^a^	1.22 ± 0.13 ^a^
4	−1.10 ± 0.08 ^a,b^	0.47 ± 0.10 ^a,b^	0.60 ± 0.05 ^a^	1.34 ± 0.09 ^a^
7	−1.13 ± 0.14 ^a,b^	0.44 ± 0.12 ^a^	0.64 ± 0.07 ^a^	1.38 ± 0.10 ^a^
10	−0.98 ± 0.15 ^a,b^	0.43 ± 0.09 ^a^	0.70 ± 0.06 ^a^	1.29 ± 0.10 ^a^
13	−1.21 ± 0.14 ^b^	0.64 ± 0.05 ^b^	1.11 ± 0.12 ^b^	1.77 ± 0.14 ^b^
16	−1.88 ± 0.16 ^c^	1.10 ± 0.05 ^c^	1.41 ± 0.08 ^c^	2.60 ± 0.11 ^c^

Groups with the same lowercase superscript letters for each column are not significantly different at the *p* < 0.05 level.
