# Correction: Stencel, R., et al. Properties of Experimental Dental Composites Containing Antibacterial Silver-Releasing Filler. *Materials* 2018, *11*, 1031

**DOI:** 10.3390/ma11112173

**Published:** 2018-11-02

**Authors:** Robert Stencel, Jacek Kasperski, Wojciech Pakieła, Anna Mertas, Elżbieta Bobela, Izabela Barszczewska-Rybarek, Grzegorz Chladek

**Affiliations:** 1 Private Practice, Center of Dentistry and Implantology, ul. Karpińskiego 3, 41-500 Chorzów, Poland; robert.stencel@op.pl; 2 Department of Prosthetic Dentistry, School of Medicine with the Division of Dentistry in Zabrze, Medical University of Silesia, pl. Akademicki 17, 41-902 Bytom, Poland; kroczek91@interia.pl; 3 Faculty of Mechanical Engineering, Institute of Engineering Materials and Biomaterials, Silesian University of Technology, ul. Konarskiego 18a, 44-100 Gliwice, Poland; wojciech.pakiela@polsl.pl; 4 Chair and Department of Microbiology and Immunology, School of Medicine with the Division of Dentistry in Zabrze, Medical University of Silesia in Katowice, ul. Jordana 19, 41-808 Zabrze, Poland; amertas@sum.edu.pl (A.M.); ebobela@sum.edu.pl (E.B.); 5 Department of Physical Chemistry and Technology of Polymers, Silesian University of Technology, 44-100 Gliwice, Poland; Izabela.Barszczewska-Rybarek@polsl.pl

In the published article, “Properties of Experimental Dental Composites Containing Antibacterial Silver-Releasing Filler” [1], we found two editing errors. The Vickers hardness was calculated according to equation 7 of reference [1], but *HV* should be in the place of *E* in reference [1]:

(7) $$\;HV=\;\frac{1.8544\times F}{d^{2}}\;$$

Moreover, *F* was the load in kgf, not in N, as it was previously described in reference [1].

We also found an editing error in Figure 6 of reference [1]. The axis should be described as “Vickers microhardness, kgf/mm^2^”, not “Vickers microhardness, MPa”.

The changes do not affect the results. The values were correct. We apologize for the inconvenience this has caused and we would like to thank the editorial office for publishing the correction. The manuscript will be updated and the original will remain online on the article webpage, with a reference to this Correction.

## Figures and Tables

**Figure 6:**
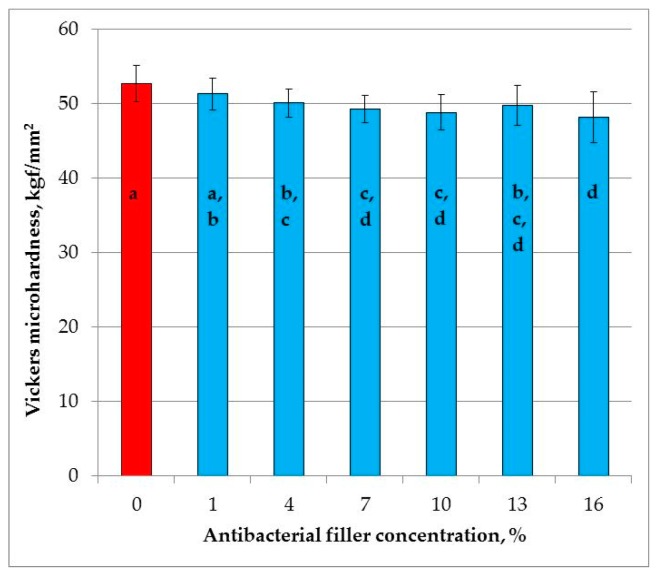
Mean Vickers microhardness values with standard deviations; different lowercase letters show significantly different results at the *p* < 0.05 level.
